# Immune Assessment of BNT162b2 m-RNA-Spike Based Vaccine Response in Adults

**DOI:** 10.3390/biomedicines9080868

**Published:** 2021-07-22

**Authors:** David San Segundo, Alejandra Comins-Boo, Juan Irure-Ventura, Mónica Renuncio-García, Adriel Roa-Bautista, Elena González-López, David Merino-Fernández, Patricia Lamadrid-Perojo, Marta Alonso-Peña, Javier Gonzalo Ocejo-Vinyals, Maria Gutiérrez-Larrañaga, Sandra Guiral-Foz, Marcos López-Hoyos

**Affiliations:** 1 Immunology Department, University Hospital Marqués de Valdecilla, 39008 Santander, Spain; david.sansegundo@scsalud.es (D.S.S.); alejandra.comins@scsalud.es (A.C.-B.); juan.irure@scsalud.es (J.I.-V.); monica.renuncio@scsalud.es (M.R.-G.); adrielantonio.roa@scsalud.es (A.R.-B.); elena.gonzalez@scsalud.es (E.G.-L.); javiergonzalo.ocejo@scsalud.es (J.G.O.-V.); maria.gutierrezl@scsalud.es (M.G.-L.); sandraguiral5@gmail.com (S.G.-F.); 2 Autoimmunity and Transplantation Research Group, Research Institute “Marqués de Valdecilla” (IDIVAL), 39011 Santander, Spain; david.merino@hotmail.com (D.M.-F.); plamadrid@idival.org (P.L.-P.); malonso@idival.org (M.A.-P.)

**Keywords:** SARS-CoV-2, vaccines, T-specific response, B-specific response, T follicular helper cells

## Abstract

Vaccine efficacy is based on clinical data. Currently, the assessment of immune response after SARS-CoV-2 vaccination is scarce. A total of 52 healthcare workers were immunized with the same lot of BNT162b2 vaccine. The immunological response against the vaccine was tested using a T-specific assay based on the expression of CD25 and CD134 after stimulation with anti-N, -S, and -M specific peptides of SARS-CoV-2. Moreover, IgG anti-S2 and -RBD antibodies were detected using ELISA. Furthermore, the cell subsets involved in the response to the vaccine were measured in peripheral blood by flow cytometry. Humoral-specific responses against the vaccine were detected in 94% and 100% after the first and second doses, respectively. Therefore, anti-S T-specific responses were observed in 57% and 90% of the subjects after the first and second doses of the vaccine, respectively. Thirty days after the second dose, significant increases in T helper 1 memory cells (*p* < 0.001), peripheral memory T follicular helper (pT_FH_) cells (*p* < 0.032), and switched memory (*p* = 0.005) were observed. This study describes the specific humoral and cellular immune responses after vaccination with the new mRNA-based BNT162b2 vaccine. A mobilization of T_FH_ into the circulation occurs, reflecting a specific activation of the immune system.

## 1. Introduction

Vaccination against SARS-CoV-2 seems to be a unique and effective way to control the pandemic outbreak. Several clinical trials have been performed to assess safety and efficacy before general use in the population [1,2,3,4]. Most of the vaccine trials focused on the clinical effect (i.e., protection against COVID-19). The production of specific SARS-CoV-2 antibodies, and specific CD4 and CD8 T cells has also been analyzed in subgroups of patients included in the clinical trials and, more recently after the conditional approval of vaccines, in specific groups of patients and populations [5]. However, such an immune response is not usually evaluated between the first and second doses of the vaccine and is not used for immune assessment of the response before clinical effect.

The average clinical efficacy range is more than 90% after two doses. However, there are differences in vaccine designs: the mRNA-1273 SARS-CoV-2 and BNT162b2 vaccines are designed based on SARS-CoV-2 Spike-mRNA, whereas Gam-COVID-Vac is a combined vector vaccine, based on rAd type 26 (rAd26) and rAd type 5 (rAd5), and ChAdOx1 nCoV-19 is a chimpanzee adenovirus-vectored vaccine [6]. At the moment of this study, no inactivated or attenuated vaccine has been approved in Europe for wide use.

The SARS-CoV-2 virus has several core units, such as nucleocapsids and the membrane, and uses the S protein to enter human cells by binding to the ACE2 receptor [7]. The described vaccines are based on an S-protein and would induce a specific response against it, while those whole-virus designed vaccines induce a wide immune response against different parts of the SARS-CoV-2 virus.

The specific-vaccine response involves germinal center activation in secondary lymphoid organs. Different cells interact in an activation as demonstrated in natural COVID-19 infection; first, T follicular helper cells (T_FH_) are activated by antigen-presenting cells with their specific antigen. Subsequently, after antigen recognition, naïve mature B cells develop to unswitched B cells before their B cell receptor’s affinity maturation to achieve the switched memory B cell (SwM) stage. Finally, follicular B cells mature to long-lived memory B cells or antibody producing-plasma cells [8]. Very recently, the induction of long-lived bone marrow plasma cells has been demonstrated up to 11 months after natural infection [9].

In order to assess the vaccine response in the community, specific antibody production against pathogens is measured. However, other immune response components could be evaluated. All of the currently approved vaccines for SARS-CoV-2 allow for assessing specific-S protein response in the vaccinated population, measuring both S-specific T cell responses and S-specific IgG antibody production [10,11].

However, to better characterize the immune response elicited by the vaccine, both cellular and humoral components should be tested. Here, we describe the differences observed in T and B lymphocytes after completing each dose of the BNT162b2 vaccine, and we focus on the induction of peripheral T follicular helper (pT_FH_) cells and the production of specific antibodies.

## 2. Materials and Methods

### 2.1. Subjects

A total of 52 healthcare workers (median age 42.5 years and interquartile range 30.5–54.2) were recruited for the study after giving written consent, of which 41 were females (78.8%). The study was addressed following the Helsinki declaration and assessed by the Regional Ethics Committee (CEIm, internal code 2020.167). All subjects had not evidenced prior COVID-19 infection with a negative PCR-specific test of SARS-CoV-2 prior immunization and were vaccinated with the same lot (EK9788) of BNT162b2 vaccine (Pfizer-BioNTech). The first dose was inoculated within 4–8 January 2021, and the second dose was inoculated within 27–30 January 2021. The samples were collected after 20 days of the first dose and after 30 days of the second dose.

### 2.2. Flow Cytometry for B and T Cell Subsets

Peripheral blood mononuclear cells (PBMCs) were obtained by Ficoll Histopaque 1077 (Sigma Aldrich, St. Louis, MI, USA) gradient centrifugation. In brief, PBMCs were freshly stained and processed following standard procedures [12,13]. The following monoclonal antibodies were used to identify the pT_FH_ and the different T lymphocyte subsets: CD45-Krome orange (KrO) clone J33, CD3-pacific blue (PB) clone UCHT1, CD4-phycoerythrin-cyanine 5.5 (PC5.5) clone 13B8.2, CD45RO-ECD clone UCHL1, (Beckman Coulter, Brea, CA, USA), CXCR3-FITC clone G025H7, CCR6 (CD196) phycoerythrin-cyanine 7 (PE Cy7) clone G034E3, and CXCR5-PE clone J252D4 (BioLegend, San Diego, CA, USA).

The different B lymphocyte subsets were identified using the following monoclonal antibodies: CD19-PC7 clone J3-119, anti-IgD-FITC clone IA6-2, and CD27-PC5.5 clone 1A4 (Beckman Coulter, Brea, CA, USA).

**Figure 1:**
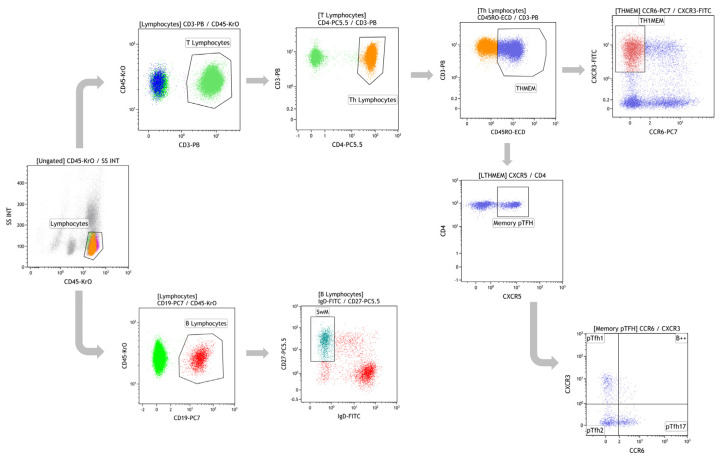
Gating strategy of T and B cell analysis explained in the text.

The gating strategy used for the different B and T cell subpopulations is depicted in Figure 1. Lymphocytes were gated based on CD45 pan-leukocyte marker. Afterwards, B and T lymphocytes were classified as CD19^+^ and CD3^+^, respectively. Within the T population, T helper (Th) cells express CD4. This subpopulation was further divided in Th memory (ThMEM) that express CD45RO surface marker and T follicular helper (T_FH_) cells with the expression of CXCR5. Within the ThMEM subset, the Th1MEM subpopulation, characterized by the expression of CXCR3 marker, was evaluated. Moreover, from the ThMEM subset, the memory pT_FH_ cells were identified based on CXCR5 expression and based on CXCR3 and CCR6, and memory pT_FH1_, pT_FH2_, and pT_FH17_ were classified [14]. Table 1 shows the phenotypic markers used to characterize each T and B cell subset shown in Figure 1.

### 2.3. SARS-CoV-2 T-Specific Response Assessment by Flow Cytometry

The procedure was validated by the Spanish Society of Immunology and based on activation-induced marker (AIM) expression after exposure with specific SARS-CoV-2 antigens [15]. Briefly, PBMCs from heparinized blood were isolated by Ficoll gradient and cultured at 10^6^/mL in TexMACS medium (MiltenyiBiotec, Bergisch Gladbach, Germany) during 24 h at 37 °C in a flat-bottom 96-well plate in 0.1% DMSO, PepTivator SARS-CoV-2 Prot S, Prot M and Prot N (1 ug/mL) and Dynabeads Human T activator CD3/CD28 (Gibco Thermo Fisher Scientific Baltics UAB, Vilnius, Lithuania) as a positive control. After incubation, the PBMCs were washed and stained with the following monoclonal antibodies: anti-CD3 (FITC) clone UCHT 1 (Inmunotech SAS Beckman Coulter, Marseille, France), anti-CD4 (APC-Vio 770) clone VIT4 (MiltenyiBiotec, Bergisch Gladbach, Germany), anti-CD8 (ECD) clone SFCI21Thy2D3 (Beckman Coulter, 737659, Brea, CA, USA), anti-CD134 (PE) clone 134-1 (Cytognos, Salamanca, Spain), and anti-CD25 (PE-Cy7) clone 2A3. The stained PBMCs samples were washed with 150 µL pf PBS and centrifuged 5 min at 1800 rpm. Finally, 2 µL of 7-Aminoactinomycin D (7-AAD) staining solution (Tonbo Biosciences, San Diego, CA, USA) and 90 µL of PBS were added before the samples were acquired on the CytoFLEX Flow Cytometer (Beckman Coulter). The results were expressed as the frequency in the AIM (CD25^+^CD134^+^) ratio obtained after specific activation to negative non-stimulated control. A ratio >3 in one of the specific SARS-CoV-2 peptides was considered positive.

**Figure 2:**
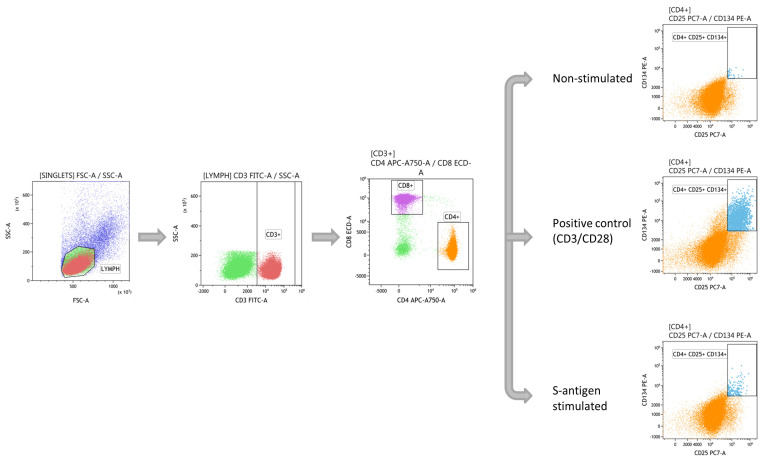
Gating strategy of the T-specific response against S-antigen of SARS-CoV-2 as explained in the text.

The gating strategy used for AIM assay is shown in Figure 2. First, lymphocytes were gated based on forward and side scatter. Then, T lymphocytes were obtained using CD3 and divided in T helper (Th) and T cytotoxic (Tc) cells by the expression of CD4 and CD8, respectively. Finally, within the CD4 subpopulation, the three conditions were displayed: non-stimulated negative control, CD3/CD28 positive control, and S-antigen stimulated.

### 2.4. SARS-CoV-2 Anti-S Antibodies Detection

The detection of IgG, IgA, and IgM antibodies against SARS-CoV-2 by ELISA was performed following the IrsiCaixa published protocol [16]. Briefly, serum samples were previously diluted 1:100 in phosphate buffer saline (PBS). Nunc MaxiSorp96-well plates (ThermoFisher Scientific, Waltham, MA, USA) were coated with optimized concentrations of 2 µg/mL of capture antibody (MA1-21315, ThermoFisher Scientific) diluted with PBS overnight at 4 °C. Coated plates were washed and blocked with PBS 1× + 1% bovine serum albumin (BSA) for two hours at room temperature. After washing the plates, antigen solution [S2 + RBD (Sino Biologicals, Wayne, PA, USA) diluted in blocking buffer] was added to one half of the plate and blocking buffer to the other half and incubated overnight at 4 °C. Serum samples were added and incubated for one hour at room temperature. Then, incubation with peroxidase-conjugated anti-IgG, -IgA, and -IgM detection antibodies (Jackson Immunoresearch, West Grove, PA, USA) was carried out for 30 min at room temperature. Bound antigen-specific antibodies were detected by adding the substrate solution. Absorbance was read at 492 nm. The specific signal associated with each sample was calculated by background subtraction as follows: OD specific signal = OD (+Ag) − OD (−Ag).

### 2.5. Statistical Analysis

Statistical analysis was performed using Graph Pad Prism software 6.0 version. The distribution of continuous variables was assessed using Kolmogorov–Smirnov/Shapiro–Wilk tests. The results were expressed as median (interquartile range (IQR)). Comparisons were based on the Kruskal–Wallis and U-Mann–Whitney tests, correspondingly. A two-sided *p*-value < 0.05 was considered statistically significant.

## 3. Results

### 3.1. T-Cell Immune Response after Vaccination

**Figure 3:**
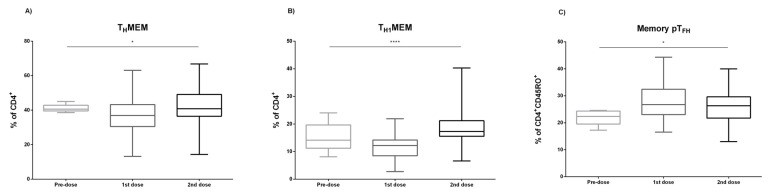
T-specific SARS-CoV-2 vaccine assessment. The frequency of memory T helper (T_H_MEM) (**A**), memory T helper 1 (T_H1_MEM) (**B**), and memory peripheral T Follicular helper (pT_FH_) (**C**), prior immunization (Pre-dose) after the first and the second doses (1st and 2nd dose, respectively) are depicted. Kruskal–Wallis test was used to compare medians in (**A**–**C**) * (*p* < 0.05) and **** (*p* < 0.0001).

**Figure 4:**
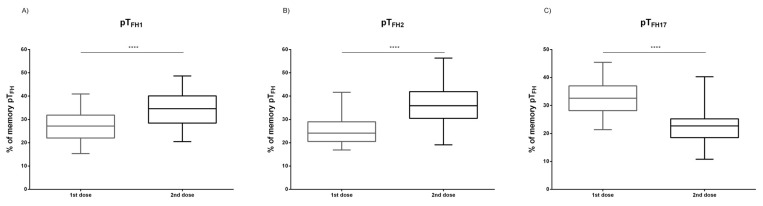
Memory peripheral T follicular helper subset assessment. The frequency of memory peripheral T Follicular helper 1 (pT_FH1_) (**A**), memory peripheral T Follicular helper 2 (pT_FH2_) (**B**), and memory peripheral T Follicular helper 17 (pT_FH17_) (**C**) after the first and the second doses (1st and 2nd dose, respectively) are depicted. U-Mann–Whitney test was used to compare medians in (**A**–**C**), **** (*p* < 0.0001).

The evaluation of the vaccine-response has been mainly based on the specific antibody production. However, specific T cells should be previously activated to induce B cell maturation in germinal centers. The frequency of total Th lymphocytes was comparable after the first and second doses. Nevertheless, the frequency of memory Th subset (CD3^+^CD4^+^CD45RO^+^) was significantly increased after the second dose, *p* = 0.008 (Figure 3A). Most of the memory Th cells were Th1 (CXCR3^+^CCR6^−^), and their proportion was also increased after the second dose, *p* < 0.0001 (Figure 3B). Within the Th cell compartment, those involved in class-switch and B cell affinity maturation in germinal centers are defined as peripheral T follicular helper cells (pT_FH_). These cells can be identified in peripheral blood as CD4^+^CD45RO^+^CXCR5^+^ cells (memory pT_FH_), and after vaccination, a significant increase of the frequency of memory pT_FH_ cells was observed, *p* = 0.032 (Figure 3C). Furthermore, a significant increase of memory pT_FH1_ and pT_FH2_ between the first and the second doses was detected (*p* < 0.0001) (Figure 4A,B). On the contrary, a significant reduction of memory pT_FH17_ was observed after the first dose (*p* < 0.0001) (Figure 4C).

### 3.2. Specific T-Cell Immune Response

**Figure 5:**
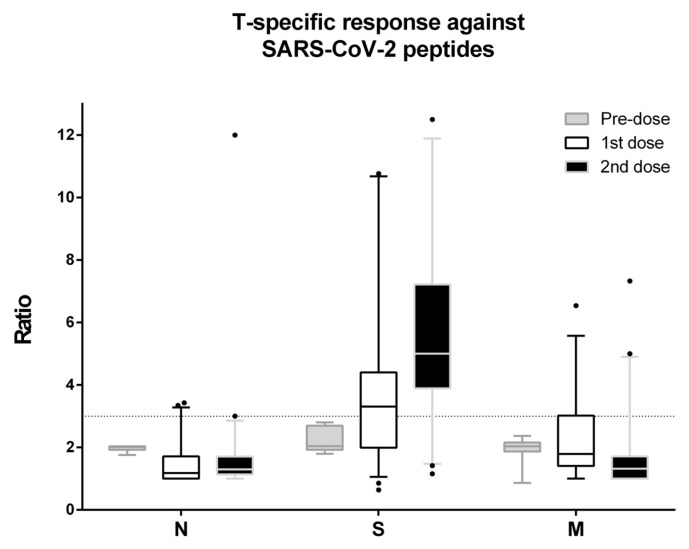
The ratio of specific CD4^+^CD25^+^CD134^+^ cells after Nucleocapside-pool (N), Spike-pool (S), and Membrane-pool (M) of SARS-CoV-2 peptide stimulation is shown. Grey (before immunization), white (after the first dose), and black (after the second dose) whisker-box plots are shown, and the cutoff to consider T-specific positive response was set at ratio >3 (dotted line).

To examine the specific T-cell response, PBMCs were exposed to anti-SARS-CoV-2 peptides using an unstimulated negative control and CD3/CD28 as positive control, as previously showed in the Material and Methods section. Thirty out of fifty-three (56.6%) subsets presented a positive specific reaction against the Spike pool peptides after the first dose. Meanwhile, after the boost, 45 out of 53 (90%) were identified as Spike-specific responses. The AIM produced with the specific S antigen after the first dose was 3.3 (1.99–4.41) and, in parallel with the frequency, significantly increased after the second dose to 5.0 (3.89–7.22), *p* < 0.0001. A cutoff of 3.0 was considered to set up a result as positive [15]. As expected, since there are no N or M antigens in the vaccine, no responses were detected for both the first and second doses (Figure 5).

### 3.3. B Cell and Antibody Response

**Figure 6:**
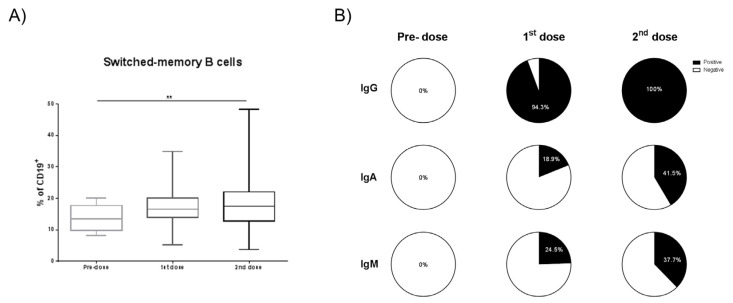
B-specific SARS-CoV-2 vaccine assessment. The frequency of switch-memory B cells (**A**) within CD19+ cells in peripheral blood, prior immunization (Pre-dose), after the first and the second doses (1st and 2nd doses, respectively) is depicted. The frequency of specific anti-SARS-CoV-2 IgG, IgA, and IgM antibodies before immunization (Pre-dose), after the first and the second doses (1st and 2nd dose, respectively) is shown (**B**). Wilcoxon test was used to median comparison in A and B, ** (*p* < 0.01).

To assess the humoral compartment of the immune response, first, the peripheral blood B cell frequency was evaluated and there was no significant difference between the frequency of B cells (CD19^+^ cells) after the first and the second doses. Second, B cell maturation stage was studied based on the surface expression of CD27 and IgD. Switched memory B cells (SwM) (CD19^+^CD27^+^IgD^+^), considered a further stage of the B cell development, were quantified. We observed a significant increase in the SwM B cell frequency after the second dose compared with the first one (*p* = 0.0054) (Figure 6A). After studying the B cell subpopulations, ELISA tests were performed following the protocol shown in the Material and Methods section to evaluate the specific serological profiles (IgG, IgA, and IgM) after BNT162b2 vaccination. Importantly, we detected IgG antibodies in 50 of 53 (94.34%), IgA in 10 (18.67%), and IgM in 13 (24.53%) of the cases after the first dose. It is noteworthy that one of the three participants did not seroconvert to IgG-specific antibodies but produced IgM antibodies. After the second boost, all volunteers developed anti-IgG antibodies (100%), and the frequency of seropositive subjects for the other two isotypes also increased: 22 (41.51%) had IgA and 20 (37.74%) had IgM (Figure 6B).

## 4. Discussion

The efficacy of vaccines in clinical trials has been described based on the frequency of infected cases and hospitalization after vaccination [17]. However, a detailed evaluation of the immune response is not fully described, and data on specific germinal center reaction cells are still scarce. In some studies, such as the Sputnik vaccine [3], Logunov et al. performed immunogenicity analysis studies in less than 10% of the subjects included in the trial.

Similarly to our findings, both cellular and humoral responses after the seasonal influenza vaccine have been studied [18], and an increase in memory T_FH_ cells after the second dose has been described. The authors observed an increase in T_FH_ cells after the seasonal flu vaccine was correlated with specific antibody development. Likewise, there is an increase in memory pT_FH1_ and pT_FH2_ cells after the second dose of the BNT162b2 vaccine. As previously described, memory pT_FH1_ cells lack the capacity to help naïve B cells in vitro while being capable of inducing memory B cells to differentiate into plasma cells [18]. Moreover, memory pTFH2 cells are able to induce naïve B cells to produce Igs and to switch isotypes through IL-21 secretion [19]. All these findings contribute to explaining the interrelation between the humoral and cellular responses after vaccination against SARS-CoV-2.

Before COVID-19, no mRNA-based vaccine had been approved for human use. However, in non-human primate models, mRNA-based influenza vaccine was assessed to elucidate cellular and humoral immune responses. This vaccine also elicited an increase of T_FH_ cells in peripheral blood after the last dose [20]. With the introduction of new mRNA-based vaccines directed to SARS-CoV-2, the main way to demonstrate sensitization against SARS-CoV-2 is based on specific antibody development. The same response level against SARS-CoV-2 after one dose in convalescents and after two doses in no-SARS-CoV-2-contact subjects was demonstrated [21]. This work is based on neutralizing antibodies, and one of the limitations in our cohort is the lack of information on neutralizing antibody production. Since our ELISA method detects anti-S-2 and –RBD antibodies, we assume that we mainly detect neutralizing antibodies [22].

First-line healthcare workers and elders were included within the first group in vaccination programs across Europe. Here we focused in healthcare workers without known comorbidities and under 65 years old. In this group an increase in SwM B, memory Th1 and memory pT_FH_ has been demonstrated. Within memory pT_FH_, three subsets have been identified, and an specific increase of both memory pT_FH1_ and pT_FH2_ at the expense of pT_FH17_ was observed. This finding could be explained because after the second dose there is an enhancement of both a cellular immune response mediated by memory pT_FH1_ [18] and an induction of germinal center reaction mediated by memory pT_FH2_ [18,19]. However, potential sensitive groups, i.e., such as patients with primary and secondary immunodeficiencies or patients under immunosuppression treatment, could also be prioritized. The patients under hemodialysis treatment have impaired immune systems [23], and a reduction in their humoral response after the BNT162b2 vaccine compared with controls has been observed [24]. Moreover, residents of long-term care facilities developed a weak humoral response after one dose of BNT162b2 [25]. However, up until now, there is a lack of data about the cellular immune response induced after vaccination in these groups of immunosuppressed patients. Unlike the clinical trial-derived data for the BNT162b2 vaccine, where the efficacy after the first dose was reported as 52%, most of the subjects included in our cohort (94%) developed anti-S-specific antibodies 20 days after the first dose. This variability in the production of antibodies could be explained due to differences in the age of the subjects included in both studies. In our study, the median age was 42.5 years (interquartile range 30.5–54.2). Therefore, the immune response is expected to be strong enough to induce a specific response with only one dose. In clinical trials, efficacy has been based on a lack of infection after the vaccination. Similarly, in our cohort, no infections were reported, and the immune response assessment has been used as a tool to test efficacy.

The efficacy of vaccinations is measured through incidence of infection, which seems to be very low (<5%) with mRNA COVID-19 vaccines [1,4]. However, in some specific disease groups, such as solid organ transplant recipients, a lack of protection against symptomatic COVID-19 has been described [26]. The measurement of anti-S SARS-CoV-2 antibodies is an objective and a biological way of assessing vaccine efficacy. Thus, a weaker humoral response in transplanted patients has been demonstrated [27,28,29,30]. The use of T cell response components in the assessment of vaccine protection is not routinely implemented, but it could be a valuable tool in specific disease or octogenarian groups.

To our knowledge, this is the first study in which peripheral blood memory T follicular helper cells have been described as relevant subpopulations implicated in the development of an immune response after SARS-CoV-2 vaccination. The higher induction of these cell subsets after the second, but not after the first, dose could help to demonstrate an effective vaccination in the weaker groups of patients indicated in the previous paragraph. A lack of response after such a second dose could be an alarm signaling to protect them and to think about the need for an additional dose to obtain an effective response.

Despite the natural course of an immune response involving the cooperation between T and B cells to generate specific cells implicated in antibody production, a humoral response has been detected before the cellular response in the present study. This finding can be explained because our study is not based on the assessment of T cell subpopulations present in secondary lymphoid organs but in the peripheral blood instead, which takes longer to be detected. On the other hand, we cannot exclude the possibility of extrafollicular responses in our cohort, although it is supposed that the production of the majority of antibodies depends on plasma cells that have undergone the somatic hypermutation and affinity maturation in the germinal center response [9].

Among the limitations of the present work, the absence of PD1 and CCR7 as markers that allow for differentiating quiescent and activated pT_FH_ cells stands out. Moreover, the identification of anti-SARS-CoV-2-specific antibodies as a dichotomous variable did not allow us to establish a correlation between the presence of these antibodies and the level of T cell subsets.

In summary, this study describes the specific immune response after vaccination with the new mRNA based BNT162b2 vaccine, measuring the production of specific antibodies and the development of a specific T cell response. Moreover, mobilization of T follicular helper and B follicular cells into the circulation occurs, with both populations implicated in the post-germinal center secondary immune response, reflecting a specific activation of the immune system after vaccination. Such an approach can help in the management of populations with some degree of immunosuppression or immunosenescence.

## Figures and Tables

**Table 1:** Phenotypic characterization of the T and B cell subsets.

Cell Subsets	Phenotypic CD Markers
**T cell subsets**	
T helper 1 (Th1)	CD45^+^CD3^+^CD4^+^CXCR3^+^CCR6^−^
Th memory (ThMEM)	CD45^+^CD3^+^CD4^+^CD45RO^+^
Th1 memory (Th1MEM)	CD45^+^CD3^+^CD4^+^CD45RO^+^CXCR3^+^CCR6^−^
Memory T follicular helper (T_FH_)	CD45^+^CD3^+^CD4^+^CD45RO^+^CXCR5^+^
Memory T follicular helper 1(T_FH1_)	CD45^+^CD3^+^CD4^+^CD45RO^+^CXCR5^+^CXCR3^+^CCR6^−^
Memory T follicular helper 1(T_FH2_)	CD45^+^CD3^+^CD4^+^CD45RO^+^CXCR5^+^CXCR3^−^CCR6^−^
Memory T follicular helper 1(T_FH17_)	CD45^+^CD3^+^CD4^+^CD45RO^+^CXCR5^+^CXCR3^−^CCR6^+^
**B cell subsets**	
Switched memory B (SwM)	CD45^+^CD19^+^CD27^+^IgD^−^
